# Hs-CRP as a biomarker for atherosclerosis progression and cardiovascular risk: a systematic review

**DOI:** 10.7717/peerj.21217

**Published:** 2026-05-04

**Authors:** Dalal A. Al-Saedi, Kholoud M. Al-Otaibi, Ahmed Alalhareth, Mohammed Aldurayhim, Abdulmajeed Alomari, Bushra Aljadani, Jumana J. Siddiqui, Fadi Alawaji, Nourh T. Almotiry, Abdurahman Alloghbi

**Affiliations:** 1 Department of Chemistry, College of Science, Princess Nourah bint Abdulrahman University, Riyadh, Saudi Arabia; 2 Department of Chemistry, Faculty of Science, Al-Baha University, Al-Baha, Saudi Arabia; 3 Najran Health Cluster, Najran, Saudi Arabia; 4 Department of Laboratory, Prince Sultan Military Medical City, Riyadh, Saudi Arabia; 5 Al Kharj Military Industries Corporation Hospital, Alkharj, Saudi Arabia; 6 Al-Ghad International Health Sciences Colleges, Jeddah, Saudi Arabia; 7 Department of Laboratory, International Medical Center, Jeddah, Saudi Arabia; 8 Qassim Health Cluster, Qassim, Saudi Arabia; 9 National Guard health affairs, Riyadh, Saudi Arabia; 10 Department of Oncology, King Khalid University, Abha, Saudi Arabia

**Keywords:** Inflammation, Cardiovascular disease, Atherosclerosis, Hs-CRP, Myocardial infarction

## Abstract

**Background:**

High-sensitivity C-reactive protein (hs-CRP) has emerged as a significant biomarker for assessing inflammatory activity in atherosclerosis, which may predict sudden death, especially in individuals with advanced atherosclerotic disease or those at high risk for acute events.

**Methods:**

This systematic review aimed to evaluate the significance of changes in hs-CRP levels over time in the population with atherosclerosis; the frequency of cardiovascular events, including mortality, stroke, and myocardial infarction; and changes in the progression of atherosclerosis. We conducted a comprehensive search of the PubMed, Scopus, Web of Science, and Embase databases up to January 2026 to identify observational and cohort studies evaluating high hs-CRP levels in patients with atherosclerosis, and to evaluate the quality of studies using the Newcastle-Ottawa Scale (NOS).

**Results:**

Our results included six studies involving baseline and follow-up hs-CRP values that showed a relationship between elevated baseline hs-CRP levels and an increased incidence of atherosclerotic progression that led to cardiovascular events. In addition, the variability in inflammation severity across different clinical events was displayed by the range of baseline hs-CRP measurements across different patient groups.

**Conclusion:**

Universal screening for hs-CRP may improve assessment and thereby reduce the risk of cardiovascular events.

## Introduction

Atherosclerosis is a chronic inflammatory condition triggered by arterial damage caused by various risk factors. Plaque instability and resulting clinical manifestations are key indicators of disease progression (Lemesle et al., 2009). According to clinical studies, the progression of atherosclerosis is associated with clinical cardiac events such as myocardial infarction (MI), heart failure, peripheral arterial disease (PAD), and unstable angina (Thygesen et al., 2018; Zhu et al., 2019). Approximately 330 million people worldwide suffer from cardiovascular diseases (CVDs), which accounts for approximately 10% of the global disease burden and 30% of annual deaths worldwide (Virani et al., 2020; Gao et al., 2024).

Inflammation plays a crucial role in the onset and progression of atherosclerosis. High-sensitivity C-reactive protein (hs-CRP), an inflammatory biomarker, has been clinically used to assess inflammatory conditions and predict vascular risk (Zhang et al., 2021; Kirkgoz, 2023). hs-CRP has attracted clinical interest as an atherosclerotic cardiovascular event associated with CVD predictors, and its levels have shown promise for predicting short- or long-term CVD in patients with CVD (Thygesen et al., 2018; Zhu et al., 2019). However, other studies have shown that its role goes beyond merely indicating inflammation; it may actively trigger the inflammatory response, affecting the function of the vascular endothelium, accelerating the development of atherosclerosis, and increasing the risk of CVD incidence and sudden death (Jordà & García-Álvarez, 2018), especially in patients with conditions such as acute coronary syndrome (ACS) and PAD (Zeller et al., 2022). A previous study evaluated the hs-CRP levels of 27,939 patients, for up to 8 years and proposed that the probability of CVD increased with an increase in CRP levels (Ridker et al., 2003). A follow-up analysis of more than 6,000 patients showed that those with higher hs-CRP levels were more likely to experience acute cardiovascular events (Danesh et al., 2004). Hence, a continuously rising hs-CRP level indicates an increased risk of atherosclerosis and CVD (Carrero et al., 2019; Sitepu & Harahap, 2020).

Although many studies have shown that hs-CRP may be a predictor of CVD, it is yet to be included in routine examinations to predict the onset or progression of CVD. Changes in hs-CRP levels over time may provide important information about the progression of atherosclerosis, stability of atherosclerotic plaques, and risk of acute events such as stroke or myocardial infarction. Furthermore, by understanding these relationships, it may be possible to improve risk assessment and optimize treatment plans for at-risk patients. We conducted cohort and observational studies in a population with atherosclerosis to determine the significance of monitoring changes in hs-CRP levels at baseline and follow-up as the disease progresses and the incidence of cardiovascular events increases.

## Materials & Methods

### Search strategy

The current protocol was registered in the International Prospective Register of Systematic Reviews PROSPERO (ID: CRD42024574466). Table S1 describes the search terms used in this review. We comprehensively searched for published studies indexed in the PubMed, Scopus, Web of Science, and EMBASE. The search was conducted on July 24, 2024, using the following search terms: (“High Sensitivity C-Reactive Protein” OR “hs-CRP”) and (“Atherosclerosis OR Atherogenesis OR “Arteriosclerotic vascular disease (ASVD”), and (“Adverse Cardiac Events”). Date, language, study type, or country of origin were not restricted during the initial search. To ensure the review remained current, the search was updated in PubMed, Scopus, and Web of Science to include studies published up to 30 January 2026 using the same search terms. EMBASE was excluded from the updated search because access to it was no longer available at the time of the update.

### Selection criteria

We included observational and cohort studies (in English) that reported the measurement of hs-CRP levels and adverse cardiac events in patients with atherosclerosis. Studies that involve individuals with atherosclerosis ≥18 years of age and have assessed hs-CRP may comprise two stages: baseline and follow-up, required to be included in eligible studies by two independent authors. Studies focusing on single-time-point measurements of hs-CRP levels were excluded, even if the study population consisted of individuals with atherosclerosis. Case-control studies, case reports, non-human studies, conference papers, and review articles were excluded. Using Rayyan software, a systematic review was conducted following the Preferred Reporting Items for Systematic Reviews and Meta-Analyses (PRISMA) 2020 guidelines (Page et al., 2021) by two blinded authors who screened the selection criteria, and disagreements were resolved with the other authors through discussion and consensus. The abstracts and titles were independently screened by Alomari and Aljadani. All disagreements were resolved by Al-Saedi and Almotiry. Full-text screening was performed by Alalhareth and Al-Saedi, with Al-Otaibi serving as a reference to resolve disputes.

### Data extraction and quality assessment

Aldurayhim completed the data extraction, and Siddiqui performed the quality assessment. The extracted data included study characteristics (author, year, study type, and duration), participant demographics (sample size, age, and sex), evaluation of hs-CRP levels at baseline and follow-up and observed outcomes. Methodological quality was assessed using the Newcastle-Ottawa Scale (Wells et al., 2011). The primary outcomes of this systematic review were MI, stroke, PAD, and angina. The secondary outcome measure was mortality.

## Results

### Literature search results

A comprehensive literature search yielded 5,448 studies. After removing 841duplicate studies, the dataset was reduced to 4,607 unique records, which underwent initial screening to assess their relevance based on the predetermined inclusion and exclusion criteria.

A significant number of studies were excluded during the screening process. Specifically, 4,283 studies were deemed ineligible for further analysis owing to their irrelevance to the research question, lack of necessary data, or failure to meet methodological standards. Finally, 324 studies were advanced to the next stage of review.

**Figure 1:**
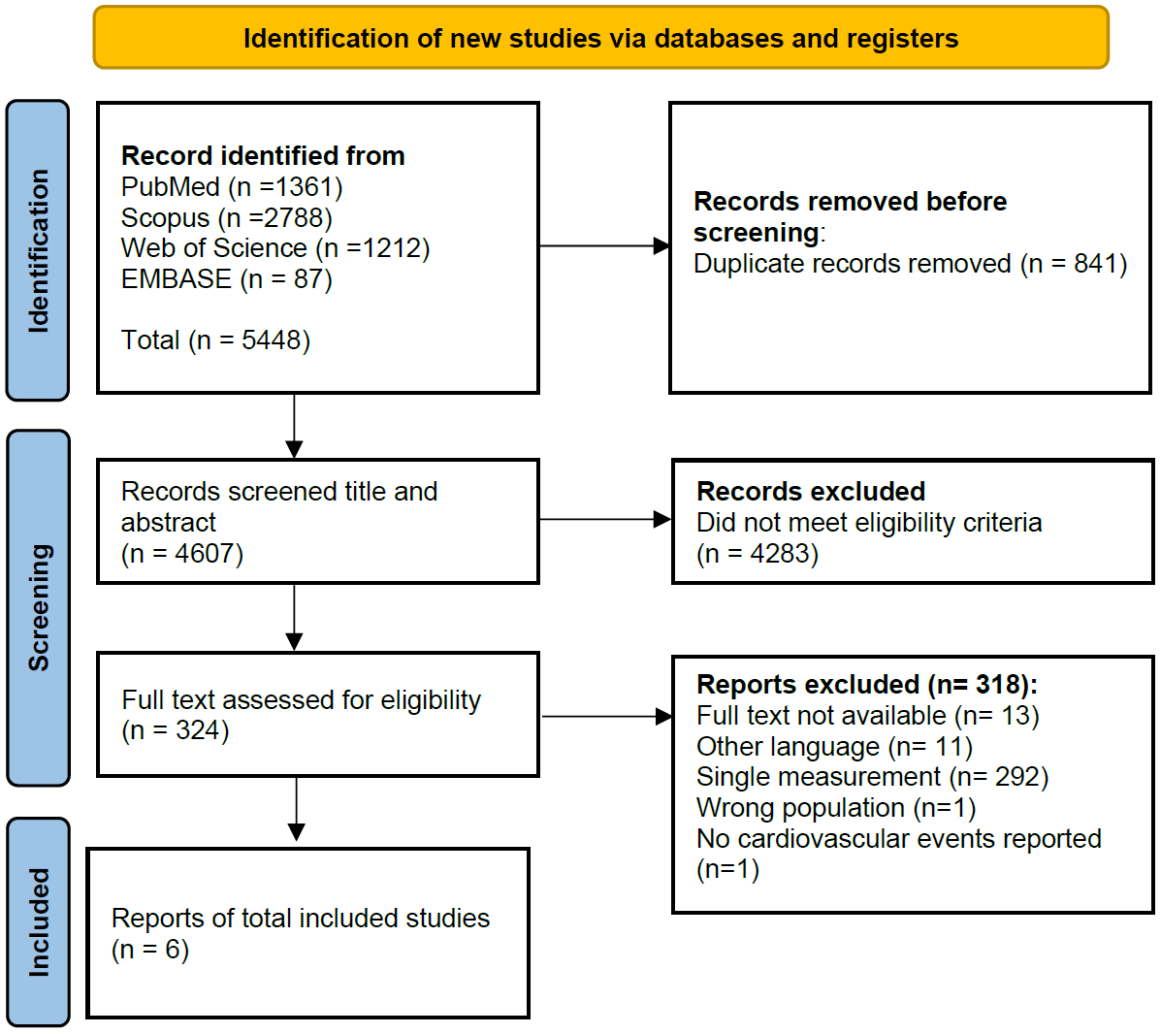
PRISMA study flow chart.

Of the 324 studies that underwent a detailed evaluation, only six met the inclusion criteria for the final analysis. These six studies were included in the synthesis, providing a foundation for the results and conclusions presented. The selection process highlights the stringent filtering applied to ensure that the included studies were of high quality and directly relevant to the research objectives. A PRISMA flow diagram summarizes selection process (Fig. 1).

### Characteristics of included studies

The characteristics of the included studies are summarized in Table 1. Each study identified a relationship between hs-CRP levels and cardiovascular outcomes in patients with coronary artery disease (CAD), ACS, and related conditions. The studies varied in design, sample size, duration, and primary outcomes; however, they shared a focus on hs-CRP as a biomarker for inflammation and its prognostic value in atherosclerosis progression or cardiovascular events such as MI, stroke, and death.

**Table 1:** Study characteristics, sample demographics, hs-CRP levels, and observed outcomes.

**Author/Year** **Study ID**	**Study type**	**Sample size, n**	**Gender** **n (%)**	**Age (mean)**	**Study duration**	Hs-CRP level (mean/median)^*^		**Outcomes observed**	**Treatment**	**Quality of study**
						**Baseline (mg/L)**	**Follow up (mg/L)**			
Feng et al. (2014)	Observational cohort study	**183 CAD**	**Male**		1 year			PP was associated with higher hs-CRP levels; lipid-rich plaques were more prevalent in the PP group, leading to increased MI. *p* < 0.001	Yes (Aspirin Statins *β*-blockers ACEI/ARB) **treatment started at baseline**	Good
		**PP group** (*n* = 78)	51 (65.38)	55.80 ± 9.43		1.24 ± 1.79	1.17 ± 1.56			
		**Non-PP** (*n* = 105)	77 (73.33)	59.18 ± 10.56		0.51 ± 1.05	0.48 ± 1.01			
Hashimoto et al. (2001)	Prospective Cohort study	179 **Early carotid atherosclerosis**	**Male** 89 (50)	62.8 ± 8.7	2 years	1.5 ± 0.21	3.2 (0.12–0.79)	Higher hs-CRP levels were associated with more rapid carotid atherosclerosis progression, increasing the risk of stroke and MI due to inflammation-driven atherosclerosis. *p* < 0.01	No	Good
Emadzadeh et al. (2013)	Observational Study	**204 ACS**	**Female**		24 h			No significant difference in baseline hsCRP values among patient groups with different levels of stenosis. *p* > 0.5	No	Good
		**MI group** (*n* = 104)	73 (70.2)	59.0 + 0.9		8.7 (6.22–11.57)	8.3 (6.6–11.35)			
		**UA group** (*n* = 100)	50 (50)	59.9 + 1.0		6.8 (4.12–10.8)	7.5 (5.5–11.2)			
		**Control** (*n* = 52)	29 (55.8)	56.8 + 0.8		2.1 (1.4–2.78)				
Vaidya et al. (2018)	Prospective nonrandomized observational study	**80 ACS**	**Male**		1 year			Colchicine therapy reduced low attenuation plaque volume and hs-CRP levels, resulting in a decreased risk of MI and stroke due to reduced plaque instability. *p* < 0.001	Yes (Colchicine) **treatment started at baseline**	Good
		40 with recent ACS (<1 month)	32 (80.0)	56.3 ± 8.9		2.95 ± 1.56	1.85 ± 0.90			
		40 controls	30 (75.0)	58.4 ± 14.2		2.64 ± 2.06	2.26 ± 1.05			
Denegri et al. (2023)	Prospective Multi-Center	**4787 ACS**	**Female**		8 years			Elevated hs-CRP is associated with a higher risk of major adverse cardiovascular events (MACE), including MI, stroke, and death. *p* < 0.001	Yes (Aspirin ACEi DAPT Statins) **treatment started at baseline**	Good
		**PAD group** (*n* = 285)	61 (21.4)	70.1 ± 10.6		14.0 ± 26.9	20.5 ± 33.6			
		**Non-PAD group** (*n* = 4502)	925 (20.5)	63.3 ± 12.4		8.9 ± 22.1	14.7 ± 31.4			
Jahn et al. (2004)	Prospective cohort study	**133 CAD**	**Male**		3 years (follow-up measurements at 6 months and a final assessment within the 3 years)			Patients with a hs-CRP level >60% of the initial level at follow-up had 80% of further cardiac over the next 3 years. *p* = 0.001	No	Good
		**have Cardiac events** (*n* = 17)	12 (70.6)	65.9		3.9 ± 1.5	2.9 ± 0.9			
		**Control** without cardio event (*n* = 113)	87 (75.0)	63.3		3.8 ± 0.9	4.1 ± 1.0			

**Notes.**

PP plaque progression ACEI/ARB angiotensin-converting enzyme inhibitors inhibitors, and angiotensin II receptor blocker UA unstable angina MI myocardial infarction CAD coronary artery disease ACS acute coronary syndromes ACEi angiotensin-converting enzyme inhibitors DAPT dual antiplatelet therapy

* According to data distribution, studies display the Hs-CRP level as mean ± SD or median and interquartile.

Findings show that most studies focused on males (Hashimoto et al., 2001; Jahn et al., 2004; Feng et al., 2014; Vaidya et al., 2018), whereas some subgroups, such as the ACS observational study, Emadzadeh et al. (2013) reported that 70% and 50% of females were classified into MI and UA groups, respectively.

Additionally, the prospective PAD cohort (Denegri et al., 2023) included 21.4% of females. Age also varied extensively, with younger populations such as the plaque progression (PP) group in the observational cohort study (Feng et al., 2014) with a mean age of 55.8 ± 9.43 years and older cohorts such as the PAD group (Denegri et al., 2023) with a mean of 70.1 ± 10.6 years.

### Hs-CRP levels and changes over time

The included studies varied in their representation of hs-CRP levels as mean or median values based on the normal distribution of their data, reporting its level as both a baseline and follow-up measure across all studies.

Overall, high hs-CRP levels are often associated with an increased risk of cardiovascular events (Hashimoto et al., 2001; Feng et al., 2014; Vaidya et al., 2018) or the progression of atherosclerosis (Jahn et al., 2004; Emadzadeh et al., 2013; Denegri et al., 2023). For instance, in the cohort study (Hashimoto et al., 2001), individuals identified with early carotid atherosclerosis who did not use cardiovascular drugs had a mean baseline hs-CRP of 1.5 ± 2.1, which increased to 3.2 mg/L at follow-up, measured using the median value. However, in the PP group in a cohort study (Feng et al., 2014), hs-CRP levels at baseline decreased slightly from 1.24 ± 1.79 mg/L to 1.17 ± 1.56 mg/L at follow-up despite the patients receiving cardiovascular drugs, such as aspirin, statins, and ACE inhibitors. Further, patients with higher hs-CRP levels were associated with more lipid-rich plaques, which worsened MI. In contrast, Vaidya et al. (2018) reported a significant reduction in hs-CRP levels with colchicine therapy, declining from 2.95 ± 1.56 mg/L at baseline to 1.85 ± 0.90 mg/L at follow-up, which is consistent with decreased plaque volume, which lowers the risk of MI and stroke because of less unstable plaque.

According to an 8-year multi-center prospective cohort, increasing hs-CRP is associated with a higher risk of major adverse cardiovascular events (MACE) (Denegri et al., 2023) in patients with PAD who received dual antiplatelet therapy (DAPT) and statins compared with those without PAD. In contrast, in observational research, where patients were followed for 24 h, no differences were observed in hs-CRP levels between patient groups with varying degrees of stenosis (Hashimoto et al., 2001).

### Study quality and risk of bias

All studies were rated as “Good” quality, indicating robustness of the methodologies and well-defined designs. However, several limitations and potential biases were noted. Feng et al. (2014) included a relatively small sample size, which may have reduced the statistical power of their findings. In contrast, Denegri et al. (2023) benefited from a large sample size; however, they faced challenges owing to the extended study duration, which introduced potential confounders such as evolving treatment protocols. Additionally, variability in study populations, follow-up durations, and intervention strategies limit the direct comparability of results across studies.

## Discussion

**Figure 2:**
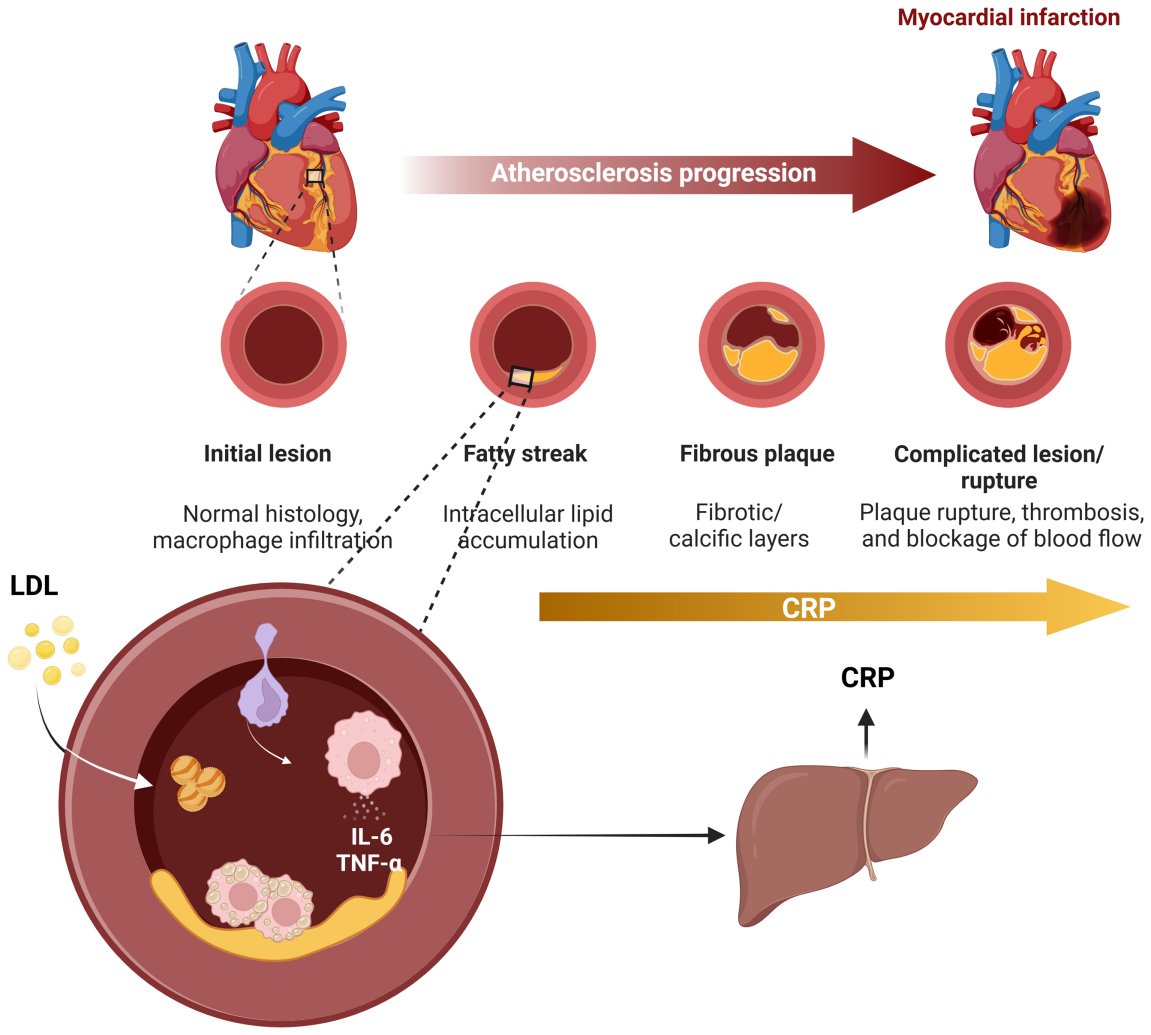
C-Reactive protein in atherosclerosis progression. The first step in the progression of atherosclerosis is the infiltration of low-density lipoprotein (LDL) into the artery wall, where it is oxidized (ox-LDL). This causes endothelial cell activation, leading to monocyte migration into the intima, where they then differentiate into macrophages, which uptake ox-LDL and transform into foam cells. Activated macrophages release IL-6, which stimulates the liver to CRP production. As plaque develops, circulating CRP levels rise, increasing the risk of cardiovascular events (Kirkgoz, 2023) [Figure created using BioRender].

Hs-CRP is an inflammatory marker that has shown potential for predicting cardiovascular risk (Fig. 2) in individuals’, both in the short and long term (Gao et al., 2024). A systematic review of six studies that investigated the relationship between hs-CRP levels and cardiovascular outcomes in patients with CAD and ACS revealed several important findings. These studies indicate a robust correlation between increased hs-CRP levels and adverse cardiovascular outcomes such as MI, stroke, and overall mortality. Second, the variation in baseline hs-CRP levels among diverse patient groups underscores the heterogeneity of inflammatory severity in various clinical scenarios, from relatively stable coronary heart disease to high-risk diseases, such as PAD. Finally, these studies highlighted the efficacy of targeted anti-inflammatory therapies, such as colchicine, in lowering hs-CRP levels and alleviating cardiovascular risk, while also highlighting the necessity for customized management strategies in high-risk populations where inflammation continues or is exacerbated despite intervention (Vaidya et al., 2018; Denegri et al., 2023).

A large cohort study included in this review, which showed that patients with PAD had significantly higher baseline and 12-month hs-CRP levels, indicating continued humoral inflammatory activity even after an acute ACS event. A higher rate of MACE, including increased 1-year mortality, recurrent MI, stroke, and limb ischemia, is associated with this inflammation. Even after controlling other risk factors, PAD remained an independent predictor of MACE (Denegri et al., 2023). In contrast, another study did not demonstrate risk association (*p*-value > 0.5), most likely due to the very short follow-up duration of only 24 h (Emadzadeh et al., 2013).

Patients with PP had persistently higher hs-CRP levels at baseline and at follow-up, indicating stronger inflammation in unstable atherosclerotic plaques. Overall, hs-CRP levels captured systemic inflammatory burden, whereas plaque characteristics offered additional complementary predictive values (Feng et al., 2014). Whereas, in patients with non-ST-elevation ACS, a decrease in hs-CRP from baseline to follow-up was observed in those who experienced no events within 6 months, whereas most events over 3 years occurred in patients whose hs-CRP levels remained above 60% of their initial levels (Jahn et al., 2004). This suggests that monitoring hs-CRP levels over time may help identify patients with CAD who are at a higher risk of future events. Notably, unlike previous studies, where reductions in hs-CRP were observed in the context of therapeutic interventions, the current study demonstrated such decreases even in the absence of any treatment, indicating that natural variations in hs-CRP may also have prognostic value.

The considerable fluctuation observed in hs-CRP levels, both at baseline and follow-up, corresponds with the results of previous research. Research has indicated that hs-CRP levels can fluctuate significantly over brief intervals, with one study documenting a within-person coefficient of variation of 46.2% (Bower et al., 2012). This intrinsic variability indicates that a single hs-CRP measurement may not consistently reflect the inflammatory condition of an individual. On the other hand, one study revealed considerable temporal fluctuations in hs-CRP levels over 24 h, with peak concentrations often occurring in the morning (Koc et al., 2010). This diurnal fluctuation may affect the interpretation of data if sampling timings are inconsistent among studies or between the baseline and follow-up measures. Likewise, recent findings highlight the need to standardise sampling protocols of inflammatory biomarkers, such as hs-CRP and IL-6, in future studies on inflammatory biomarkers (Gorey et al., 2025). Although inflammation and hyperlipidemia work together to initiate and develop atherosclerosis, risk factors such as blood pressure and low-density lipoprotein (LDL) cholesterol are the most controlled screening factors for diagnosis and follow-up of patients. The utility of hs-CRP as an independent biomarker has certain constraints. Research indicates that approximately one-third of individuals with increased CRP levels may be reclassified as having normal CRP levels upon further testing (Bower et al., 2012). In a cross-sectional investigation of patients with dyslipidemia, hs-CRP levels were found to be independently linked with triglycerides and BMI and to be strongly correlated with the severity of atherosclerosis. This implies that, in addition to traditional risk markers such as LDL, inflammation that is determined *via* hs-CRP adds predictive value (Swastini et al., 2019). Although useful as a prognostic marker, a recent review emphasizes that hs-CRP should be interpreted alongside other clinical factors and that repeated measurements may further improve risk prediction (Fatima et al., 2025). In contrast, individuals with elevated hs-CRP levels (>2 mg/L) have elevated lipoprotein(a) Lp(a) levels, which are linked to an increased risk of atherosclerotic cardiovascular disease (ASCVD) (Zhang et al., 2021). However, Lp(a) levels may independently predict the risk of ASCVD in individuals with a history of ASCVD (Fichtner et al., 2024). Therefore, further research is needed to determine the ideal Lp(a) and hs-CRP thresholds for targeted interventions.

However, limitations such as varying sample sizes, study durations, and intervention strategies across the included studies may have affected the direct comparability of the results and underscore the need for more standardized, large-scale trials to further elucidate the role of hs-CRP in cardiovascular outcomes.

## Conclusions

This review revealed a strong and consistent association between elevated hs-CRP levels and adverse cardiovascular outcomes, including MI, stroke, and mortality. These findings emphasize that it is a predictive biomarker for the progression of atherosclerosis and increases the incidence of cardiovascular events. Future research should focus on including hs-CRP as an indicator in routine diagnostic processes to enhance early diagnosis and intervention.

##  Supplemental Information

10.7717/peerj.21217/supp-1 Supplemental Information 1 PRISMA checklist

10.7717/peerj.21217/supp-2 Supplemental Information 2 Medical Subject Headings (MeSH) used for literature search

10.7717/peerj.21217/supp-3 Supplemental Information 3 Rationale

## Additional Information and Declarations

### Data Availability

The following information was supplied regarding data availability: This is a Systematic review/Meta-analysis.
