# Supplementary material for: Hs-CRP as a biomarker for atherosclerosis progression and cardiovascular risk: a systematic review
Source: PeerJ. 2026 May 4;14:e21217. doi: 10.7717/peerj.21217 (PMC13151924; doi:10.7717/peerj.21217)
Supplement: Supplemental Information 2 [file peerj-14-21217-s002.docx]

**Table S1: MeSH Terms used for literature search**

| **Key words** | **MesH terms** |
| --- | --- |
| **hsCRP** | (“C Reactive Protein” OR “hs-CRP” OR “High Sensitivity C-Reactive Protein” OR “high-sensitivity CRP” OR “C-Reactive Protein High-Sensitivity” OR “High sensitivity CRP” OR “high C-reactive protein”) |
| **Atherosclerosis** | (Atheroscleroses OR Atherogenesis OR “Arteriosclerotic vascular disease (ASVD)” OR Arteriosclerosis OR “carotid intima-media thickness CIMT” OR “coronary artery calcium score” OR CACS)) |
| **Adverse Cardiac Events** | (“Cardiovascular event” OR “cardiovascular disease (CVD)” OR “Cardiac Events” OR “Adverse Cardiac Events” OR “Major Adverse Cardiac Events” OR “Heart disease” OR “coronary artery disease (CAD)” OR “coronary heart disease (CHD)” OR “Myocardial Infarctions” OR “Heart Attack” OR “Myocardial Infarct” OR “Cardiovascular Stroke” OR “Heart failure (HF)” OR “congestive heart failure (CHF)” OR “Cardiac Failure” OR “Heart Decompensation” OR “congestive cardiac failure (CCF)” OR “Myocardial Failure” OR “Peripheral Artery Disease (PAD)” OR “transient ischemic attack” OR TIAs OR “Carotid Circulation Transient Ischemic Attack” OR “Sudden Cardiac Death” OR “Cardiac Arrests, Sudden” OR Strokes “Vascular Endothelium” OR Atheroma OR “Atherosclerotic Plaque”)) |
