# Supplementary material for: Hs-CRP as a biomarker for atherosclerosis progression and cardiovascular risk: a systematic review
Source: PeerJ. 2026 May 4;14:e21217. doi: 10.7717/peerj.21217 (PMC13151924; doi:10.7717/peerj.21217)
Supplement: Supplemental Information 3 [file peerj-14-21217-s003.docx]

**Systematic Review Rationale**

This review is aimed at researchers working in biochemistry, cardiovascular biology, and atherosclerosis. Although high-sensitivity C-reactive protein (hs-CRP) has been investigated extensively, its value as a prognostic marker for atherosclerosis development is still unclear. Our review fills this gap by assessing the prognostic value for atherosclerotic progression and cardiovascular events by measuring baseline and follow-up hs-CRP values. The findings will help us to use hs-CRP as a universal screening tool, which may enhance evaluation and reduce the risk of cardiovascular events.
